# Survival of Probiotic Bacterial Cells in the Upper Gastrointestinal Tract and the Effect of the Surviving Population on the Colonic Microbial Community Activity and Composition

**DOI:** 10.3390/nu16162791

**Published:** 2024-08-21

**Authors:** Marlies Govaert, Chloë Rotsaert, Chelsea Vannieuwenhuyse, Cindy Duysburgh, Sophie Medlin, Massimo Marzorati, Harry Jarrett

**Affiliations:** 1 ProDigest BV, Technologiepark 82, 9052 Ghent, Belgium; marlies.govaert@prodigest.eu (M.G.); chloe.rotsaert@prodigest.eu (C.R.); chelsea.vannieuwenhuyse@prodigest.eu (C.V.);; 2 Heights, Department for Research and Development, London W1D 2LG, UK; sophie@citydietitians.co.uk (S.M.); harry@yourheights.com (H.J.); 3 Center of Microbial Ecology and Technology (CMET), Ghent University, Coupure Links 653, 9000 Ghent, Belgium

**Keywords:** probiotics, survival, gastrointestinal tract, administration strategies, delayed-release capsule, gut microbiota

## Abstract

Many health-promoting effects have been attributed to the intake of probiotic cells. However, it is important that probiotic cells arrive at the site of their activity in a viable state in order to exert their beneficial effects. Careful selection of the appropriate probiotic formulation is therefore required as mainly the type of probiotic species/strain and the administration strategy may affect survival of the probiotic cells during the upper gastrointestinal (GIT) passage. Therefore, the current study implemented Simulator of the Human Microbial Ecosystem (SHIME^®^) technology to investigate the efficacy of different commercially available probiotic formulations on the survival and culturability of probiotic bacteria during upper GIT passage. Moreover, Colon-on-a-Plate (CoaP™) technology was applied to assess the effect of the surviving probiotic bacteria on the gut microbial community (activity and composition) of three human donors. Significantly greater survival and culturability rates were reported for the delayed-release capsule formulation (>50%) as compared to the powder, liquid, and standard capsule formulations (<1%) (*p* < 0.05), indicating that the delayed-release capsule was most efficacious in delivering live bacteria cells. Indeed, administration of the delayed-release capsule probiotic digest resulted in enhanced production of SCFAs and shifted gut microbial community composition towards beneficial bacterial species. These results thus indicate that careful selection of the appropriate probiotic formulation and administration strategy is crucial to deliver probiotic cells in a viable state at the site of their activity (distal ileum and colon).

## 1. Introduction

The collection of bacteria, archaea, and eukarya colonizing the gastrointestinal tract (GIT) is termed the gut microbiota. Such microbiota has an important role in lipid, cholesterol and glucose metabolism, and in maintaining energy and immune homeostasis, playing a fundamental role in human health and disease prevention [1]. Alterations in gut microbiome composition and function (gut dysbiosis) characterized by decreases in beneficial bacteria (such as *Bifidobacteriaceae* and *Lactobacillaceae* strains) have been associated with a number of adverse health outcomes, including obesity, diabetes, cognitive impairment, and depression [2,3], whereas supplementation with probiotics has been shown to increase gut microbiome diversity, leading to improvements in body composition, cognitive function, and mood [4,5,6].

Once engrafted in the colonic environment, probiotic cells can modulate the colonic fermentation processes through the direct production of their respective metabolites or indirectly by generating end-products which can be used as cross-feeding substrates for other colonic bacteria. As a result, an alteration in the community composition can occur by stimulation and/or downregulation of certain (groups of) bacterial species. Moreover, the modulatory effect of probiotics on the functioning and composition of the colonic microbiota could result in a modulatory effect on the host itself which is commonly referred to as the host–microbiota interaction. For example, probiotic supplementation has been shown to restore gut barrier integrity [7,8,9,10,11].

Oral drug delivery is often preferred over other routes, including intravenous, intramuscular, intranasal, and intradermal administration, due to the sustainable and controllable nature of delivery [12]. Despite the advantage of the ease of administration, many challenges are associated with oral administration of probiotic cells. Namely, the probiotic of interest needs to survive the harsh stomach environment, which is generally characterized by a low pH and the presence of gastric enzymes. Upon entering the small intestinal environment, the probiotic cells are, amongst others, exposed to relatively high bile salt concentrations, which can be detrimental to many microbial species. As such factors can lead to inactivation of the administered microbial cells prior to arrival at their site of activity (distal ileum and colon), the applied administration method should be carefully assessed [13]. Probiotic cells can, for example, be administered as a liquid formulation or a powdered formulation which needs to be suspended in water prior to consumption. Other colon-targeted release strategies include encapsulation and compression into tablets, which provide easy administration, long-term stability/shelf life, and high consumer acceptance. These capsules or tablets can also be coated to protect the content from the harsh stomach and/or small intestinal environment until the shell is degraded. Depending on the type of coating, disintegration of the capsule or tablet can, for example, be triggered by changes in environmental pH or colon-specific enzymatic degradation. As a result, a targeted release of the probiotic cells can be envisioned, preventing inactivation of the cells during passage through the upper GIT [14]. As yeast cells are generally more resistant towards acidic environments than bacterial cells, selection of the most optimal administration method is crucial to ensure optimal delivery of the bacterial cells [15].

In vitro models that simulate the human (upper) GIT are considered as an important tool in research focusing on oral drug delivery as these models help to study the behavior and stability of drugs while applying different delivery methods [16]. The Simulator of the Human Microbial Ecosystem (SHIME^®^) can, for example, be adapted to simulate the physiological conditions of the stomach and small intestine, serving as an interesting model for capsule dissolution characteristics and evaluation of probiotic survival [16,17]. Moreover, the effect of the surviving probiotic cells in the colonic environment can subsequently be assessed by means of short-term colonic batch fermentations using Colon-on-a-Plate™ (CoaP) technology [18].

Therefore, the aim of this study was to utilize the SHIME^®^ technology platform to assess bacterial culturability and survival during passage through the upper GIT using four commercially available probiotic formulations, each containing a specific mixture of probiotic species and using a specific administration strategy. The administration strategies of interest were (i) a liquid formulation, (ii) a powdered formulation, (iii) a standard (not acid-resistant) capsule formulation, and (iv) a delayed-release (DR, acid-resistant) capsule formulation. Subsequently, a short-term colonic simulation was executed for the probiotic formulation which showed the highest survival rate during the upper GIT passage. More specifically, the effect of the surviving cells on the gut microbial community activity and composition was examined, either alone or in combination with different prebiotic compounds (i.e., β-glucan, galacto-oligosaccharides (GOS), and fructo-oligosaccharides (FOS)), using CoaP technology.

## 2. Materials and Methods

### 2.1. Test Products

In this study, the SHIME^®^ technology platform (ProDigest and Ghent University, Ghent, Belgium) was used to examine the effect of different commercially available probiotic formulations on the culturability and survival of bacterial cells during passage through the upper GIT. More specifically, a liquid formulation, powdered formulation, standard capsule, and DR capsule were assessed. In addition, a blank control incubation without probiotic administration was included. The exact probiotic composition of the different commercially available formulations is summarized in Table 1, while more information regarding the excipients is presented in Supplementary Table S1. Due to the limitations of the system, half of a single in vivo intake dose was used for the following test products during the in vitro simulations: ‘liquid’, ‘powder’, and ‘capsule’. For the ‘capsule DR’ test product, the full in vivo dose was administered. As yeast cells are generally more resistant towards the harsh environmental conditions encountered during the upper GIT passage, this study specifically focused on the bacterial species incorporated within each of these commercially available formulations. All probiotic formulations were tested before the end of the shelf-life period was reached.

### 2.2. Upper Gastrointestinal Tract Simulation under Fasted Conditions

The simulation of the upper GIT under fasted conditions was performed according to Marzorati, et al. [16]. In brief, a double-jacketed reactor, simulating the stomach and small intestine digestion conditions as a function of time, was maintained at 37 °C under continuous magnetic stirring (300 rpm; 2mag, München, Germany). The stomach digestion was simulated with a 45 min incubation in a gastric fluid (76 mL, pH 2), containing KCl 0.66 g/L (Chem-lab, Zedelgem, Belgium), NaCl 3.63 g/L (VWR Chemicals, Leuven, Belgium), and mucin 3.95 g/L (Carl Roth, Karlsruhe, Germany), 0.4 mL of lecithin (3.4 g/L, Carl Roth), and 3.6 mL pepsin (10 g/L, Carl Roth). Continuous pH control was performed by a Senseline pH meter F410 (ProSense, Oosterhout, The Netherlands) and an automatic pump dosage of HCl (0.5 M, Chem-lab) or NaOH (0.5 M, Chem-lab) to keep the pH constant at 2. At the start of the simulation, the liquid and powder formulations were added directly to the gastric fluid, while the capsules were incorporated in the reactor with specifically designed sinkers for capsule dissolution studies (ProSense). The capsule sinkers were positioned in the middle of the liquid volume, perpendicular to the stirring direction. At the end of the stomach incubation, small intestinal fluid containing 35.2 mL pancreatic juice (NaHCO_3_ 2.6 g/L (Chem-lab), Oxgall 4.8 g/L (Difco™ Oxgall, BD, Erembodegem, Belgium), and pancreatin 1.9 g/L (Merck Life Sciences, Darmstadt, Germany)), 2.15 mL trypsin (10 g/L, Carl Roth), as well as 2.7 mL chymotrypsin (10 g/L, Carl Roth) were added to the reactor to initiate the small intestinal incubation. Under continuous stirring (300 rpm), the small intestinal pH was gradually increased from 2 to 6.5 and maintained over a 27 min period, simulating the duodenal incubation. Subsequently, the pH was increased in a stepwise manner until a value of 7.5 was reached over the course of 63 min, simulating the jejunal environment. Finally, the pH remained constant at 7.5 during a 90 min ileal incubation. All experiments were performed in biological triplicate (*n* = 3) to account for biological variability.

### 2.3. Colon-on-a-Plate™

The colonic environment was mimicked by CoaP technology (ProDigest), a miniaturized version of a short-term batch fermentation model. The system makes use of deep well plates in order to incubate small volumes of colonic medium, fecal inoculum, and the pre-digested test product. As the highest survival rate at the end of the upper GIT simulation was obtained for a capsule DR, this specific commercial probiotic formulation was selected for the colonic incubations, where the pre-digested test product was either tested alone or in combination with different prebiotic ingredients (Table 2). Each condition was tested in single using a fecal inoculum of three different adult human donors in order to account for inter-donor variability.

The CoaP was conducted using 4.2 mL of colonic medium, containing host-and diet-derived substances including peptone (Oxoid Ltd., Hampshire, UK), yeast extract (Oxoid Ltd.), and L-cysteine (Merck Life Sciences). This colonic medium was mixed with 2 mL upper GIT digesta (collected at the end of the ileal incubation) and 0.7 mL fecal inoculum. The inocula were derived from freshly collected samples of three healthy (BMI = 18.5–24.9; not diagnosed with a specific disease linked to an altered gut microbiome) adult (age 28–34 y) individuals who had not been treated with antibiotics six months prior to sample collection. The samples were cryopreserved with a modified version of the cryoprotectant developed by Hoefman, et al. [19] for storage, before addition to the colonic incubations. Prebiotic fibers were supplemented as described in Table 2. Colonic digestion conditions were maintained at 37 °C, in an anaerobic atmosphere, and under continuous mild shaking (90 rpm; IKA, Staufen, Germany) for 48 h.

### 2.4. Study Endpoints

#### 2.4.1. Visual Scoring of Capsules during the Upper Gastrointestinal Incubation

Visual inspection of the integrity of the capsules was performed at different sampling times, i.e., the start of the upper GIT incubation and the end of the stomach (ST end), duodenum (DUO end), jejunum (JEJ end), and ileum (ILE end) incubations by the same researcher. The capsules were scored as follows: (1) the capsule intact; (2) the capsule damaged, but almost all probiotic content (>75%) remains in the capsule; (3) the capsule damaged, but about 50% of the probiotic content remains in the capsule; (4) the capsule damaged, with 25% of the probiotic content still inside the capsule; (5) the capsule shell still visible, but all the product content is released; and (6) the capsule is fully dissolved.

#### 2.4.2. Bacterial Culturability during the Upper Gastrointestinal Incubation

For each probiotic formulation, spread plating was used to determine the culturable bacterial population density before and after the upper GIT passage. Samples were therefore collected at the end of the small intestine incubation (ILE end). Moreover, the test product was analyzed to determine the initial baseline values. The liquid test product was analyzed without any sample preparations, while the powder and capsule formulations (i.e., capsule contents) were suspended in anaerobic phosphate buffer saline (PBS) solution prior to sample collection. More specifically, 6 g powder was dissolved in 80 mL anaerobic PBS, while the content of a single capsule was dissolved in 30 mL anaerobic PBS. In case the encapsulated formulation was not fully disintegrated at the end of the upper GIT simulation, the remaining capsule residue was also analyzed by suspending the remaining capsule content in 10 mL anaerobic PBS prior to sample collection. For each sample, a ten-fold dilutions series was prepared in anaerobic PBS and subsequently transferred to Petri dishes containing BHI (Brain Hearth Infusion) agar (Thermo Fisher Diagnostics, Dardilly Cedex, France) for enumeration of the total anaerobic bacterial population. BHI agar plates were therefore incubated anaerobically at 37 °C for 48 h.

The number of colony-forming units (CFUs) is reported as average CFU/reactor ± stdev (*n* = 3). Moreover, the culturable population densities obtained at the ILE end and the corresponding baseline values obtained for the product as such were used to calculate the culturability rate, which is reported as average % ± stdev (*n* = 3). The reported values at the ILE end are based on the total culturable population densities retrieved by the ILE end, meaning that both the released fraction and the fraction which was still present in the capsule residue (if applicable) were considered.

#### 2.4.3. Bacterial Survival during the Upper Gastrointestinal Incubation Assessed through PMA-Based qPCR

Bacterial survival was tested by propidium monoazide (PMA)-based quantitative real-time polymerase chain reaction (qPCR) analysis. Samples collected at different stages of the experiment for the stomach (i.e., ST end), small intestine (i.e., DUO end, JEJ end, ILE end), and capsule residue (if applicable) were therefore assessed. Moreover, the products as such were again analyzed using a similar sample preparation as indicated in Section 2.4.2. For all samples, a 1:1 (*v*/*v*) dilution of the sample in anaerobic PBS was mixed with 1.25 µL PMAxx™ dye (20 mM, VWR International Europe, Leuven, Belgium) and incubated for 5 min under constant shaking (500 rpm; Thermo Fisher Scientific Bvba, Merelbeke, Belgium) conditions in a dark environment. Subsequently, the samples were centrifuged at maximal speed (18,327× *g*; Hettich, Kirchlengern, Germany) for 30 s, placed in the PhAST blue PhotActivation System (GenIUL, Barcelona, Spain), a LED-active Blue system (GenIUL), for 15 min, and centrifuged for 10 min at 13,000× *g*. The supernatant was immediately removed, and DNA was isolated as described before by Boon, et al. [20], with modifications described in Duysburgh, et al. [21]. The qPCR analyses were performed with primers for total bacteria [UNI-F (5′-GTGSTGCAYGGYYGTCGTCA-3′) and UNI-R (5′-ACGTCRTCCMCNCCTTCCTC-3′)] [22] for the liquid, powdered, and capsule formulation, and *Bifidobacterium* spp. [Bif243F (5′-TCGCGTCYGGTGTGAAAG-3′) and Bif243R(5′-CCACATCCAGCRTCCAC-3′)] [23] and *Lactobacillus* spp. [F_lacto_05 (5′-AGCAGTAGGGAATCTTCCA-3′) and R_lacto_04 (5′-CGCCACTGGTGTTCYTCCATATA-3′)] [24] for the DR capsule, using a QuantStudio 5 Real-Time PCR system (Applied Biosystems, Foster City, CA, USA) with program conditions previously described in Van den Abbeele, et al. [25,26].

The viability results are reported as average 16S rRNA copies/reactor ± stdev (*n* = 3). Moreover, the viable population densities obtained at the ILE end and the corresponding baseline values obtained for the product as such were used to calculate the survival rate, which is reported as average % ± stdev (*n* = 3). The reported values at the ILE end are based on the total culturable population densities retrieved by the ILE end, meaning that both the released fraction and the fraction which was still present in the capsule residue (if applicable) were considered.

#### 2.4.4. Fermentic and Metabolic Activity of Gut Microbiota under Colonic Conditions

The microbial activity assessment was based on samples collected at the end of the colonic incubation (i.e., following 48 h of incubation), except for the gas pressure, which was also measured at the beginning of the colonic incubation. The gas pressure change between 0 and 48 h of incubation was thus of particular interest. The gas pressure was measured using a handheld pressure indicator (WIKA, Lawrenceville, GA, USA) with a transmitter (WIKA). pH measurements were performed using a Senseline pH meter F410 (ProSense). Quantitative analysis of the short-chain fatty acids (SCFAs) was performed using a Shimadzu GC2030 gas chromatograph (Shimadzu Benelux, Wemmel, Belgium) with an autosampler and flame-ionization detector. The isolation of SCFAs was performed via a GC-FID method described by De Weirdt, et al. [27], after applying a diethyl ether extraction (with addition of 2-methyl hexanoic acid as internal standard). Lactate production was assessed with the Enzytec™ kit, according to the manufacturer’s instructions (R-Biopharm, Darmstadt, Germany). Finally, ammonium was determined colorimetrically (AQ300 Discrete Analyzer, Seal Analytical, Rijen, The Netherlands) using the indophenol blue spectrophotometric method. Results are reported as pH (-), gas pressure (kPa), SCFAs (mM), lactate (mM), and ammonium-N (mg/L). Each of the measurements was conducted in single repetition.

#### 2.4.5. Microbial Composition of Gut Microbiota under Colonic Conditions

At the end of the colonic simulations, samples were collected to assess the effect of the different test conditions on the gut microbial community composition. DNA was extracted from the pellet of 1 mL sample (obtained upon centrifugation for 5 min at 9000× *g*) as previously described [20]. The original genomic DNA extracts were diluted in DNase/RNase/protease-free water (Thermo Fisher Scientific Bvba) to obtain a concentration of 50 ng/µL and 30 µL was sent for 16S-rRNA gene profiling by LGC Genomics GmbH (Berlin, Germany). Library preparation and sequencing were performed on an Illumina MiSeq platform with v3 chemistry with 341F (5′-CCTACGGGNGGCWGCAG-3′) and 785Rmod (5′-GACTACHVGGGTATCTAAKCC-3′) primers, adapted from Klindworth, et al. [28]. The proportional phylogenetic information (%) was combined with a quantification of the total number of cells via flow cytometry. Samples for flowcytometry were diluted in Dulbecco’s Phosphate-buffered Saline (DPBS) (Sigma-Aldrich, Bornem, Belgium) and stained with 0.01 mM SYTO24 (Life Technologies Europe, Merelbeke, Belgium) for 15 min at 37 °C. Subsequently, samples were analyzed on a BD Accuri C6 Plus (BD Biosciences, Erembodegem, Belgium) using the high flow rate. Bacterial cells were separated from the medium debris and signal noise by applying two threshold values, i.e., a primary FSC-H threshold of 200 and a secondary FL-1 threshold of 700. Flowcytometry results were analyzed using FlowJo, version 10.9.0.

Shifts in community composition between the different test conditions were determined by means of Discriminant Analysis of Principal Components (DAPC) and differential abundance analysis (see Section 2.5). The community composition results were thus presented by means of DAPC and volcano plots.

### 2.5. Statistical Methods

For the upper GIT simulations, statistically significant differences between the number of viable or culturable microbial cells were determined in between each sampling point and its preceding one to demonstrate changes as a function of time, with separate statistical tests being performed for each administration strategy. Moreover, significant differences between the different administration strategies were determined based on the values obtained at the end of the upper GIT incubation (i.e., the ILE end). It should be noted, however, that the latter comparison was based on a full in vivo dose being tested for all administration strategies. The differences for all data discussed were significant with a confidence interval of 95%, as demonstrated using a Student’s *t*-test. Statistical differences were indicated based on the following classification criteria: ‘***’ if 0 ≤ *p*-value < 0.001, ‘**’ if 0.001 ≤ *p*-value < 0.01, and ‘*’ if 0.01 ≤ *p*-value < 0.05. Raw *p*-values are included.

For the colonic simulations, statistically significant differences between the blank control and all other test conditions in terms of metabolic activity were assessed based on the results obtained at the end of the colonic incubation. Only for the gas pressure, the change between 0 and 48 h of incubation was taken into account. The differences for all data discussed were significant with a confidence interval of 95%, as demonstrated using a Student’s *t*-test. The same classification criteria were used as for the upper GIT simulations, with raw *p*-values being included. In terms of community composition, whether or not a specific treatment affected the overall community composition was assessed using DAPC and differential abundance analysis. DAPC joins two analysis methods to assess effects on the population structure. In this approach, sequence data are transformed using principal component analysis (PCA), and clusters are subsequently identified with discriminant analysis (DA) [29]. The DA aims to maximize among-group variation and minimize within-group variation. In this approach, the groups (treatments) used in the DA were a priori defined. Differential abundance analysis was performed using treeclimbR analysis. This analysis was performed on relative abundance data (obtained by total sum scaling) to identify the taxa most likely to explain differences between conditions. As mentioned before, the outcome of this statistical analysis was plotted in a volcano plot, which is constructed by plotting the negative logarithm (base 10) of the raw *p*-value on the y-axis and the logarithm (base 2) of the fold change (FC; blank control vs treatment) on the x-axis. Bacterial enrichments exceeding a fold change of 4 (log_2_(4) = 2 on the x-axis) as compared to the blank control as a reference condition are considered biologically relevant by consensus. The cut-off for statistical significance is set at a raw *p*-value of 0.05 (or −log_10_(0.05) = 1.3 on the y-axis). The obtained scatterplot thus classifies taxa into four different categories based on abundance [30], i.e., (i) not significant and not biologically relevant (−2 < log_2_FC < +2, and −log_10_(*p*-value) < 1.3), (ii) biologically relevant, but not statistically significant (log_2_FC < −2 or log_2_FC > +2, and −log_10_(*p*-value) < 1.3), (iii) statistically significant, but not biologically relevant (−2 < log_2_FC < +2, and −log_10_(*p*-value) > 1.3), and (iv) biologically relevant and statistically significant (log_2_FC < −2 or log_2_FC > +2, and −log_10_(*p*-value) > 1.3).

## 3. Results

### 3.1. Bacterial Culturability and Stability during Upper Gastrointestinal Passage under Fasted Experimental Conditions

**Figure 1:**
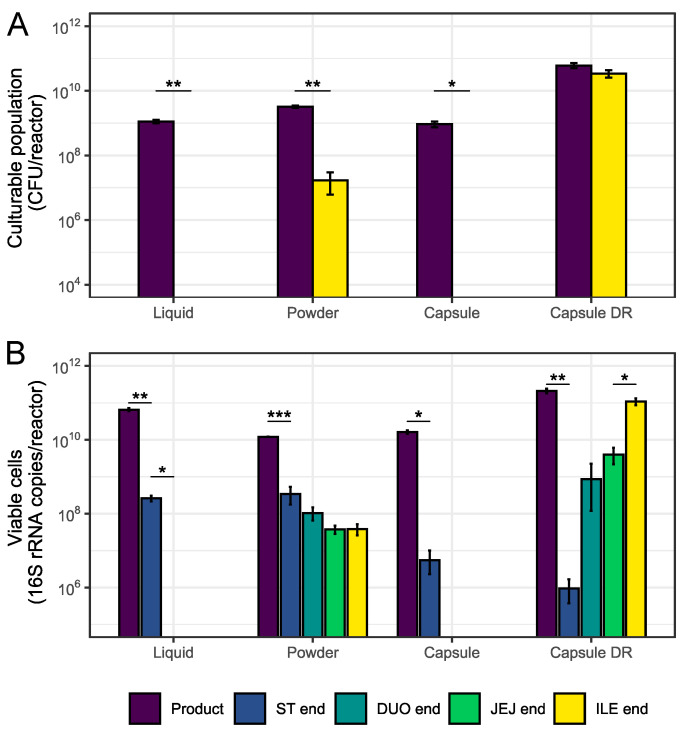
Average culturable ((**A**) CFU/reactor) and viable ((**B**) 16S rRNA copies/reactor) population densities ± stdev (*n* = 3) of the different test products during passage through the upper GIT under fasted conditions. Samples were collected from the product itself (Product) and at different time points during passage through the stomach (ST end (viability only)) and small intestinal region (DUO end (viability only), JEJ end (viability only), and ILE end). For the values at ILE end, both the released fraction and encapsulated fraction in the remaining capsule residue (if any) are considered. Statistical differences from the preceding time point are indicated based on the following classification criteria: ‘***’ if 0 ≤ *p*-value < 0.001, ‘**’ if 0.001 ≤ *p*-value < 0.01, and ‘*’ if 0.01 ≤ *p*-value < 0.05.

**Figure 2:**
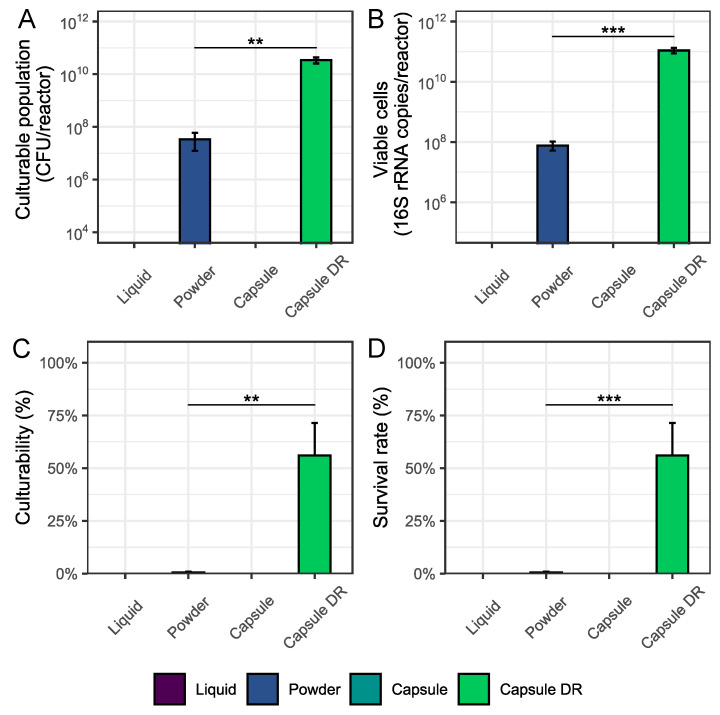
Average culturable ((**A**) CFU/reactor) and viable ((**B**) 16S rRNA copies/reactor) population densities and the corresponding average culturability ((**C**) %) and survival ((**D**) %) rates ± stdev (*n* = 3) of the different test products obtained at the end of the upper GIT simulation under fasted conditions (=ILE end). The culturability and survival rates are calculated by dividing the culturable and viable population densities obtained at ILE end by the corresponding culturable and viable population densities measured for the product as such. Statistical differences between the different test products are indicated based on the following classification criteria: ‘***’ if 0 ≤ *p*-value < 0.001, ‘**’ if 0.001 ≤ *p*-value < 0.01, and ‘*’ if 0.01 ≤ *p*-value < 0.05.

During the first part of this study, four different commercially available probiotic formulations, each representing a different administration strategy, were subjected to transit through the upper GIT under fasted conditions. For the encapsulated formulations, visual inspection of the capsule integrity was performed at different time points to monitor disintegration of the capsules over time. These scores are presented in Table 3. The culturability and viability of the bacterial cells were also monitored during the upper GIT passage and these results are shown in Figure 1. Figure 2 allows for a better comparison between all four probiotic formulations by focusing on the culturable and viable bacterial population densities, and corresponding culturability and survival rates, measured at the end of the upper GIT simulation (=ILE end). As depicted in Section 2, a ½ in vivo dose was used for the liquid, powder, and standard capsule formulation due to limitations of the system. For the results presented in Figure 2, administration of a complete in vivo dose was assumed, meaning that the values obtained for the liquid, powder, and standard capsule formulation were multiplied with a factor 2. This can, however, only be done while assuming that the bacterial response would not be affected by an alteration of the test dose with a factor 2.

Visual scoring of the capsules indicated that the standard capsule content was completely released into the environment by the end of the gastric phase (ST end), while only 50% of the capsule DR content was released by the end of the small intestinal phase (the ILE end). The probiotic cells incorporated within the standard capsule were thus entirely exposed to the harsh environmental conditions encountered during the gastric and small intestinal passage, while most of the capsule DR content remained protected.

There were also significant differences in bacterial culturability and viability between the different probiotic formulations. Specifically, during the passage through the upper GIT, the culturable population density decreased to below the limit of quantification (LOQ) for the liquid formulation (*p* = 0.005) and the standard capsule (*p* = 0.013). The culturability of the powder formulation also significantly decreased (*p* = 0.002), but a distinct fraction of the administered cells remained culturable by the ILE end. For the capsule DR, a minor decrease in the culturable population density was observed at the end of the upper GIT simulation; however, this was not statistically significant when compared to the baseline product. For the viable population density, the gastric passage resulted in significantly lower values as compared to the baseline product for the liquid product (*p* = 0.003), the powder (*p* = <0.001), the standard capsule (*p* = 0.021), and the capsule DR (*p* = 0.006). For the former three formulations, this means that a decrease in the viable cell population was noted due to exposure of the bacterial cells to the harsh gastric environment. The capsule DR, on the other hand, remained largely intact during the gastric passage, so the relatively low viable population density observed upon administration of this specific formulation was likely mainly the result of only a small portion of the probiotic strains being released at this stage. For the liquid formulation, the viable cell concentration further decreased throughout the small intestinal phase, reaching significance (*p* = 0.011) and levels below the LOQ by the end of the duodenal phase. Similarly, for the standard capsule, viable cell levels decreased to levels below the LOQ by the end of the duodenal phase. For the powder, on the other hand, only small gradual decreases were observed during the passage through the small intestinal phase, with a fraction of the administered bacterial cells reaching the end of the ileal phase in a viable state. Finally, a gradual increase in the viable population density throughout the small intestinal phase was observed upon administration of the capsule DR, with a large fraction of the capsule content reaching the end of the ileal phase in a viable state. This gradual increase in the bacterial viability was associated with a gradual disintegration of the capsule during the upper GIT passage, which largely protected the cells from the low pH values and high bile salts levels encountered in the simulated gastric and small intestinal environment.

Based on Figure 2, it was concluded that administration of the capsule DR resulted in the highest culturable (3.39 × 10^10^ ± 9.35 × 10^9^ CFU/reactor) and viable (1.09 × 10^11^ ± 2.19 × 10^10^ 16S rRNA copies/reactor) bacterial cell densities at the end of the upper GIT simulation, with the concentration of culturable and viable cells being significantly higher as compared to the powder formulation (1.69 × 10^7^ ± 1.42 × 10^7^ CFU/reactor (*p* = 0.003) and 3.80 × 10^7^ ± 1.19 × 10^7^ 16S rRNA copies/reactor (*p* = 0.001), respectively). Upon plotting the culturability and survival rates, it was observed that 56.0 ± 15.4% and 51.7 ± 10.4% of the respective original culturable and viable bacterial populations were retrieved by the end of the upper GIT simulation upon administration of the capsule DR. For the powder formulation, these percentages were lower than 1%.

### 3.2. Colonic Incubation of the Upper GIT Digesta from the Delayed-Release Capsule Using the Colon-on-a-Plate™ Technology

In the second part of this study, the formulation with the highest probiotic survival rate during the upper GIT passage (i.e., the capsule DR) was selected for further investigation at the colonic level. More specifically, the effect of the surviving probiotic cells on the gut microbial community activity and composition was assessed using CoaP technology, either with or without additional prebiotic supplementation.

**Figure 3:**
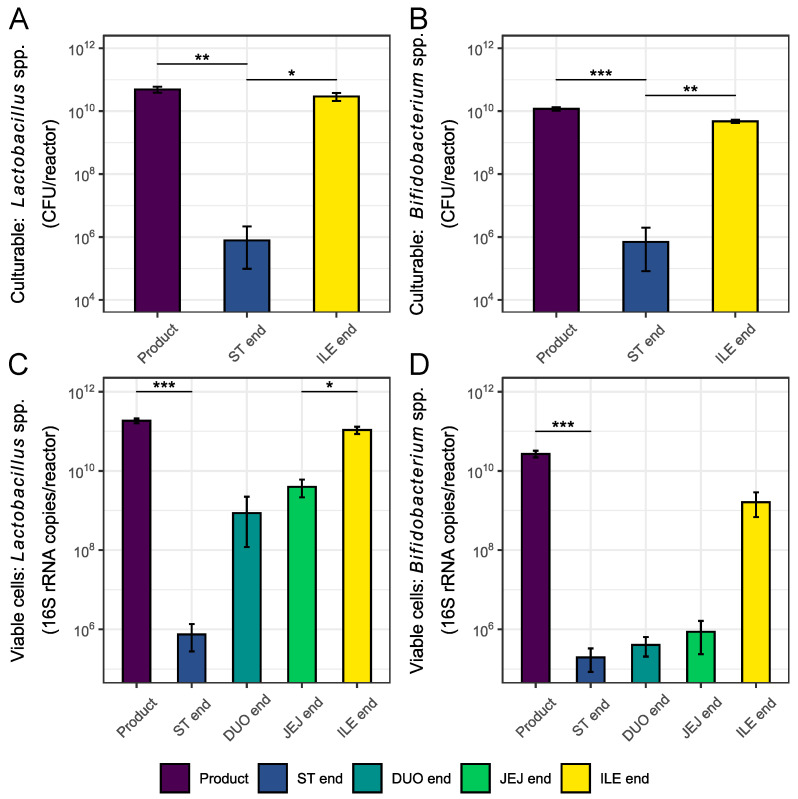
Average culturable ((**A**,**B**) CFU/reactor) and viable ((**C**,**D**) 16S rRNA copies/reactor) *Lactobacillus* spp. (**A**,**C**) and *Bifidobacterium* spp. (**B**,**D**) population densities ± stdev (*n* = 3) observed during passage through the upper GIT under fasted conditions upon administration of the capsule DR. Samples were collected from the product itself (i.e., Product) and at different time points during passage through the stomach (i.e., ST end (viability only)) and small intestinal region (i.e., DUO end (viability only), JEJ end (viability only), end ILE end). For the values at the ILE end, both the released fraction and encapsulated fraction in the remaining capsule residue are considered. Statistical differences from the preceding time point are indicated based on the following classification criteria: ‘***’ if 0 ≤ *p*-value < 0.001, ‘**’ if 0.001 ≤ *p*-value < 0.01, and ‘*’ if 0.01 ≤ *p*-value < 0.05.

Prior to execution of the short-term colonic simulations, it was important to know the composition of the selected upper GIT digesta at the family level. These results could then later on be linked with any of the potentially observed probiotic effects during the subsequent short-term colonic simulations. As indicated in Section 2, the culturability and viability of *Lactobacillus* spp. and *Bifidobacterium* spp. were separately assessed through the use of specific primers. The results shown in Figure 3 indicate that both the culturable and viable *Lactobacillus* spp. and *Bifidobacterium* spp. populations obtained at the end of the gastric incubation were significantly lower as compared to the corresponding values measured for the product as such, which was linked with the limited capsule degradation during the gastric passage. A (gradual) increase was subsequently observed during the small intestinal passage due to a continuous further release of the capsule content. By the end of the ileal phase, the measured culturable *Lactobacillus* spp. and *Bifidobacterium* spp. population densities were equal to 2.92 × 10^10^ ± 9.05 × 10^9^ and 4.76 × 10^9^ ± 5.13 × 10^8^ CFU/reactor, respectively. The viable population densities were equal to 1.07 × 10^11^ ± 2.11 × 10^10^ for *Lactobacillus* spp. and 1.62 × 10^9^ ± 8.76 × 10^8^ for *Bifidobacterium* spp. The environmental conditions encountered during the upper GIT passage thus mainly had a negative impact on the viability and culturability of the *Bifidobacterium* spp. cells.

**Figure 4:**
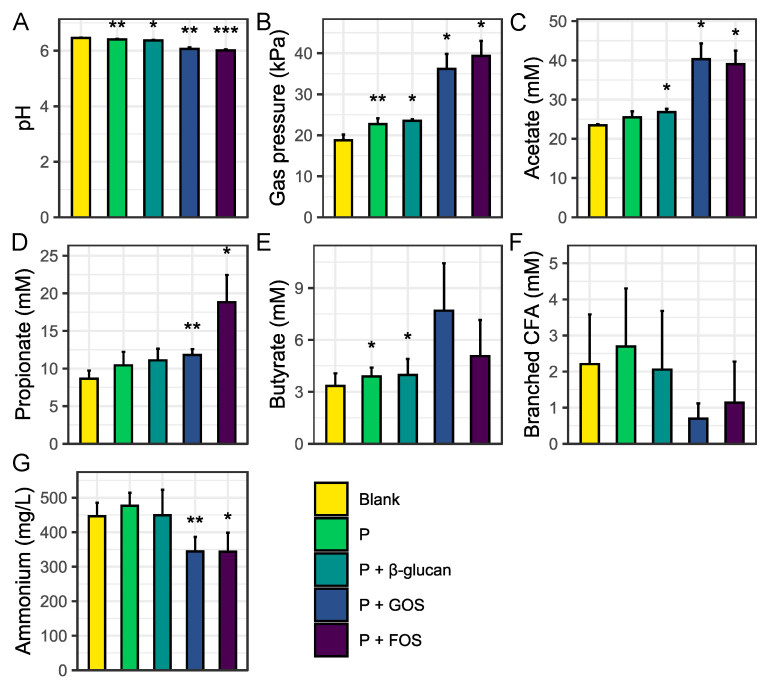
Average pH ((**A**) -), gas pressure ((**B**) kPa), acetate ((**C**) mM), propionate ((**D**) mM), butyrate ((**E**) mM), branched CFA ((**F**) mM), and ammonium ((**G**) mg/L) concentrations ± stdev (*n* = 3) measured by the end of the 48 h colonic incubations for the different treatment conditions as compared to the blank control, with error bars as a measure of interindividual variation. P = probiotic digesta of capsule DR. GOS = galacto-oligosaccharides and FOS = fructo-oligosaccharides. Statistical differences between the blank control and any of the treatment conditions are indicated based on the following classification criteria: ‘***’ if 0 ≤ *p*-value < 0.001, ‘**’ if 0.001 ≤ *p*-value < 0.01, and ‘*’ if 0.01 ≤ *p*-value < 0.05.

The metabolic activity results obtained following 48 h of colonic incubation are presented in Figure 4. As depicted in Section 2, (production) levels of the various metabolic markers in the blank control provided baseline levels to which any metabolic effect in the other test conditions was compared. First, it was observed that the administered probiotic cells significantly affected the gut microbial activity across the three donors, as observed by a reduction in the intestinal pH (*p* = 0.005), a stimulated SCFA production (*p* = 0.093 (acetate), 0.086 (propionate), and 0.045 (butyrate)), and an increased gas production (*p* = 0.006) as compared to the control. Indeed, acetate, propionate, butyrate, and bCFA levels increased upon administration of the probiotic digesta, though only reaching significance for butyrate (*p* = 0.045). Similar results were obtained when the probiotic digest was co-supplemented with β-glucan. Specifically, the intestinal pH reduced (*p* = 0.010), the gas pressure increased (*p* = 0.042), and the production of SCFAs led to significantly elevated acetate (*p* = 0.012) and butyrate (*p* = 0.037) levels as compared to the blank. The combination of the probiotic digest with either GOS or FOS induced even stronger effects in terms of saccharolytic fermentation, i.e., stronger reductions in the intestinal pH were noted for the combination with GOS (*p* = 0.004) or FOS (*p* = <0.001) as well as an increased gas (*p* = 0.024 and *p* = 0.012, respectively), acetate (*p* = 0.019 and *p* = 0.013, respectively), and propionate (*p* = 0.008 and *p* = 0.020, respectively) production. Moreover, co-supplementation with GOS or FOS also resulted in a significantly reduced ammonium production as compared to the blank control (*p* = 0.007 and *p* = 0.011, respectively), which was not observed for the probiotic digest alone or in combination with β-glucan. Lactate was not detected for any of the test conditions, which was likely linked with the establishment of efficient cross-feeding interactions. 

**Figure 5:**
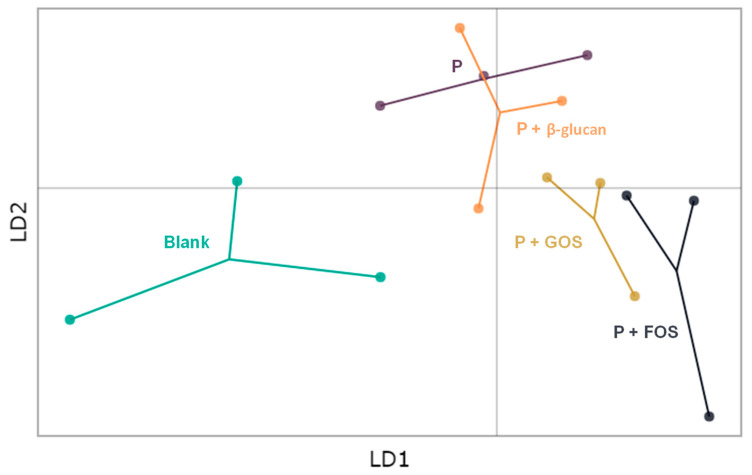
Discriminant analysis of principal components (DAPC) to show differences in community composition (beta-diversity) between the various conditions 48 h after start of the short-term colonic incubation. Each color represents a different condition (treatments or control) and each dot represents one donor (*n* = 3). LD1 and LD2 are Linear Discriminants. P = probiotic digesta of capsule DR. GOS = galacto-oligosaccharides and FOS = fructo-oligosaccharides.

**Figure 6:**
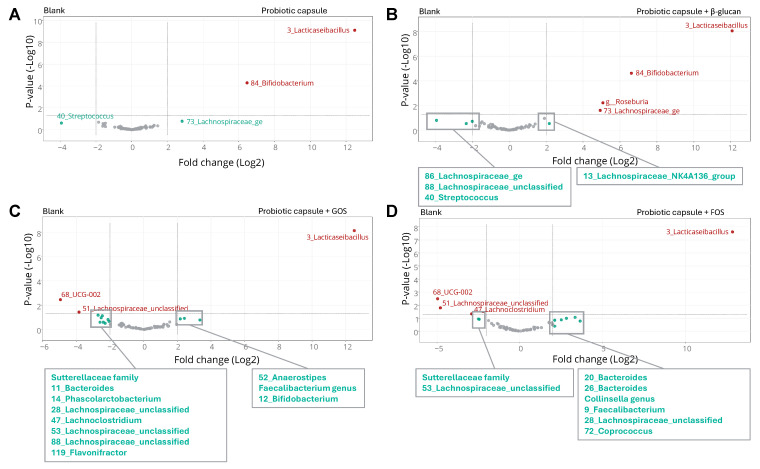
Volcano plots, obtained through differential abundance analysis (treeclimbR), indicating differences in luminal microbial community composition between treatment ((**A**) probiotic digesta, (**B**) probiotic digesta + β-glucan, (**C**) probiotic digesta + GOS, and (**D**) probiotic digesta + FOS) and blank across donors at the end of the 48 h incubation period. GOS = galacto-oligosaccharides and FOS = fructo-oligosaccharides. The obtained scatter plot classifies taxa into four categories based on abundance in compared treatments: (a) not significant and not biologically relevant (grey), (b) biologically relevant, but not statistically significant (green), (c) statistically significant, but not biologically relevant (blue), and (d) biologically relevant and statistically significant (red).

In terms of community composition, the segregation of the different test conditions from the blank in the DAPC plot (Figure 5) indicated that the overall community composition was altered upon administration of the probiotic digesta, either with or without prebiotic co-supplementation. The impact of β-glucan on top of the probiotic digesta was mild as a high degree of similarity in terms of community composition was observed with the probiotic digesta alone. In contrast, co-supplementation of GOS and FOS resulted in a stronger alteration of the overall community composition as compared to the blank control, with (slightly) different effects being observed for both prebiotic compounds. The differential abundance analysis results (Figure 6) indicated that addition of the probiotic digesta, either alone or in combination with any of the prebiotic compounds, resulted in a biologically and statistically significant higher abundance of *Lacticaseibacillus* OTU3 as compared to the blank control, with fold changes of +12.5 (*p* < 0.001 for all treatments vs. blank). As OTU3 was only encountered in the treatment conditions and not in the blank, OTU3 likely represented a multiple of the *Lactobacillus* agents present in the probiotic digest. Similarly, the abundance of *Bifidobacterium* OTU84 was significantly higher upon addition of the probiotic digesta, either alone (*p* < 0.001) or in combination with β-glucan (*p* < 0.001), as compared to the blank. Fold changes of +6.5 were observed. As OTU84 was again only encountered in the treatment conditions and not in the blank, it is likely that OTU84 represents the *Bifidobacterium* strains in the probiotic digesta. Interestingly, the probiotic digesta alone did not result in any statistically significant bacterial enrichments across the three donors, other than the OTUs representing the probiotic agents, suggesting a mild impact on the overall microbial community composition. However, co-supplementation of β-glucan resulted in additional enrichments versus the blank. More specifically, a significant enrichment of the genus *Roseburia* and of OTU73, an unknown *Lachnospiraceae* OTU, was obtained (*p* = 0.006, log FC = 5.1). Co-supplementation of GOS resulted in a biologically relevant enrichment of *Bifidobacterium* OTU12 (*p* = 0.167, log FC = 3.3), *Anaerostipes* OTU52 (*p* = 0.122, log FC = 2.4), and the *Faecalibacterium* genus (*p* = 0.135, log FC = 2.2), as compared to the blank. Finally, co-supplementation of FOS resulted in the biologically relevant enrichments of several *Bacteroides* OTUs (i.e., OTU20 (*p* = 0.086, log FC = 3.3) and OTU26 (*p* = 0.102, log FC = 2.8)), the *Collinsella* genus (*p* = 0.166, log FC = 3.6), *Coprococcus* OTU72 (*p* = 0.143, log FC = 2.1), *Faecalibacterium* OTU9 (*p* = 0.133, log FC = 2.5), and of OTU28 (*p* = 0.410, log FC = 2.1), an unclassified *Lachnospiraceae* species, as compared to the blank.

## 4. Discussion

A number of important findings were reported in this study. Firstly, the capsule DR formula led to significantly higher culturable and viable cell populations following passage through the human GIT, when compared to powder, liquid, and standard capsule formulations. Secondly the capsule DR formula was able to significantly influence human gut microbial activity, following passage through the human digestive tract. Interestingly, it was also reported that *Bifidobacterium* strains may be less stable during human digestion when compared to *Lactobacillus* strains.

A plethora of commercial oral probiotic formulations is currently available on the market, which makes it difficult for both consumers and (medical) experts to select the most optimal formulation [31]. The efficacy of probiotic formulations depends on many different aspects. Firstly, a sufficiently high number of probiotic cells should be able to survive the harsh environmental conditions encountered during the upper GIT passage. The acidic gastric pH and the relatively high bile salts and pancreatic enzyme levels in the small intestine are the most important elements to overcome [13,32]. Secondly, the surviving probiotic cells should be able to proliferate within the colonic environment, meaning that the administered cells should, for example, be able to withstand competition for nutrients and engraftment sites with the autochthonous gut microbial community [7]. Finally, the presence of the probiotic cells in the colonic environment should govern health-promoting effects. As summarized, a variety of health-promoting effects have been associated with probiotic intake, including (i) the prevention of intestinal infections, cardiovascular diseases, and cancer development, (ii) an increased tolerance to lactose, and (iii) an improved immune system. These beneficial effects can be induced in a direct or indirect way. A direct effect means that the beneficial effect is caused by the administered cells themselves (e.g., by the production of beneficial metabolites). An indirect effect means that the presence of the probiotic cells, for example, results in an enhanced abundance of specific autochthonous gut microbiota, which in turn might produce beneficial metabolites [7,8,11]. The latter phenomenon is typically referred to as a mechanism of cross-feeding interactions, in which primary metabolites produced by one type of bacterial species serve as substrates for another type of bacterial species in order to produce secondary metabolites [10]. 

### 4.1. Survival of the Probiotic Cells during Upper GIT Passage

Probiotic survival during the upper GIT passage depends on many different factors, including the selection of the microbial species, the applied dose, and the administration strategy [31].

Careful selection of the probiotic species is of high importance as the sensitivity of the cells towards the encountered gastric and small intestinal environmental conditions is species- and/or strain-dependent. Yeast cells are typically more acid-resistant than bacterial cells [33], but major differences can still be observed between different yeasts or bacterial species. In this trial, we report that the *Lactobacillus* spp. cells incorporated within the capsule DR formulation proved to be more sensitive than the *Bifidobacterium* spp. cells to the environments encountered during human digestion. To our knowledge, this is the first study to report such findings, although the findings of strain-specific differences are supported by previous trials, for example by the in vitro study of Cuffaro, et al. [34], which indicated that *Barnesiella intestinihominis* cells were more resistant towards the acidic stomach environment than *Bacteroides coprocola* cells. Such finding have important implications for future probiotic formulations and gut-microbiome-targeted public health policies aimed to improve health and reduce disease risk.

While dose-response effects were not examined within the present study, it should be acknowledged that the dose of the probiotic cells can play an important role. According to previous research, a daily consumption of 10^8^–10^9^ probiotic cells would be required to achieve any clinically significant effects [31]. Typically, probiotic cells are overdosed to mitigate any viability reduction during the product storage. In addition, if in vitro and/or in vivo tests indicated that a large fraction of the administered cells will be inactivated during the upper GIT passage, higher doses will be applied in order to entail the delivery of a distinct number of viable cells at the site of their activity (i.e., distal ileum and colon). Unfortunately, it is very hard to determine a minimum number of viable probiotic cells required to achieve health-promoting effects in the colonic region as this minimum number of viable cells will depend on the administered probiotic species (or even strain) and the environmental conditions encountered in the colonic region, with the latter showing a high inter-individual variability [35]. 

The selected administration strategy also largely affects probiotic survival. Probiotic cells are often supplemented as part of a dairy product (e.g., in a yoghurt or yoghurt drink). The food matrix can provide a protective effect during the upper GIT passage and the undigested food compounds can be used as nutrients in the colonic environment. Moreover, the presence of the food matrix induces a fed state, which is typically reflected by higher gastric pH values. The study of Feldman and Barnett [36], involving 365 healthy adult humans, reported average fasted gastric pH values ranging between 2.07 and 2.97. Fed gastric pH values showed a gradual decrease as a function of time, with pH values above 6.0 being observed at the moment of food intake, and pH values around 2.0 being observed 2h after food intake [37,38]. Nevertheless, the presence of a food matrix, or fed administration, is not always sufficient to protect probiotic cells from inactivation. The study of Fredua-Agyeman and Gaisford [31], for example, showed a strong reduction in the viable probiotic cell density when Yakult, a commercially available probiotic drink, was exposed to a simulated gastric environment at a pH-value of 1.6. When probiotic cells are administered as a powder or aqueous formulation, the excipients can again govern a protective effect during the upper GIT passage and serve as nutrients in the colonic environment. This protective effect was not observed within the present study as the survival rates of the powdered and liquid formulation were <1%. It should be noted, however, that the environmental pH values in the current in vitro study were actively controlled, meaning that fixed pH intervals were used for all test conditions in order to simulate a fasted state. A potential increase in the environmental pH upon administration of the liquid or powder formulation was thus not taken into account. Finally, the use of encapsulated formulations can also help to overcome (strong) probiotic inactivation during the upper GIT passage by protecting the cells against the low gastric pH and the relatively high bile salts and pancreatic enzyme levels encountered during the small intestinal passage. Within the currently presented study, only the delayed-release capsule formulation governed a protective effect, which was linked with capsule disintegration mainly occurring in the distal part of the small intestine. Bile salts can be toxic for bacterial cells, especially at the relatively low pH values encountered during the duodenal passage [39]. However, along the longitudinal axis of the small intestine, an increase in the environmental pH as well as a reduction in both the bile salts and pancreatic enzyme levels is observed [39]. A delayed release of the probiotic cells is therefore often favored over an immediate release. Delayed-release capsule formulations were previously also favored over standard capsule or powdered formulations in the studies of Marzorati et al. [40], Dodoo et al. [41], and Marzorati et al. [16]. The in vitro study of Dodoo et al. [41], for example, showed a 6-log reduction in the viable *Lactobacillus* spp. cell population density during the gastric passage when administered as a powdered formulation. However, when these *Lactobacillus* spp. cells were administered as delayed-release capsule formulations, recovery percentages above 88% were obtained by the end of the small intestinal phase. Therefore, the present study findings concur with previous evidence indicating that delivery of probiotics by means of a delayed-release capsule allows optimal delivery of the bacteria to the active sites. This provides important guidance for clinicians and consumers, to not only consider the probiotic strain and quantity in the supplement, but also to consider the vehicle of delivery to ensure that viable concentrations of the bacteria can be delivered and proliferate to induce the desired clinical benefits.

### 4.2. Prolifiration of the Probiotic Cells in the Colonic Environment and the (Indirect) Production of Beneficial Metabolites

Following the upper GIT passage, the surviving cells should be able to proliferate within the colonic environment or, in other words, be able to compete with the autochthonous gut microbial community for engraftment sites and/or nutrients. The short-term colonic simulations in the presence of the probiotic digest from the delayed-release capsule formulation indicated that the administered *Lactobacillus* spp. were able to colonize the gut microbial environment as indicated by a significant enrichment of OTU3. In addition, the significantly enhanced abundance of OTU84 indicated that successful colonic engraftment was obtained by (some of) the administered *Bifidobacterium* spp. The former observation was valid for the probiotic digest alone or in combination with the three different prebiotics, while the latter observation was only valid for the probiotic digest alone or in combination with β-glucan. Co-supplementation with GOS and FOS thus likely caused an alteration to the colonic environment which had a negative impact on the abundance of the administered *Bifidobacterium* spp. cells. The latter is not in line with previous in vivo and in vitro studies examining the effect of GOS and FOS on the gut microbial community composition [42,43,44]. The in vivo study of Liu et al. [42] reported an increased *Bifidobacterium* spp. abundance in adult humans following intake of FOS and GOS. In infants, intake of GOS and GOS+FOS also resulted in an increased *Bifidobacterium* spp. abundance [43]. The in vitro study of Marzorati et al. [44] also showed an increased abundance of *Bifidobacterium longum*, *Bifidobacteriaceae*, *Bifidobacterium adolecentis*, and *Bifidobacterium ruminantium* upon administration of GOS. Differences between the current in vitro study and previously performed in vivo and in vitro studies can be explained by the fact that these previous studies focused on the autochthonous *Bifidobacterium* spp. abundance, while OTU84 detected in the current study was linked with the administered allochthonous *Bifidobacterium* spp. Indeed, when the probiotic digest was co-supplemented with GOS, a biologically relevant increase in OTU 12, an autochthonous *Bifidobacterium* species, was observed. The study of Dong et al. [45] also indicated that the growth extent of *Bifidobacterium* spp. in the presence of GOS and FOS depended on the type of strain and the type of prebiotic fiber. A one-on-one comparison between different studies is thus not straightforward. The significantly increased *Bifidobacterium* spp. abundance observed for the probiotic digest alone or in combination with β-glucan can be considered as beneficial since previous studies indicated that members of the *Bifidobacteriaceae* family have, amongst others, the potential to (i) prevent and/or treat colorectal cancer (CRC), (ii) reduce symptoms of inflammatory bowel disease (IBD), and (iii) treat diarrhea [46].

Focusing on the autochthonous microbial community, it was observed that co-supplementation with β-glucan induced an enhanced *Roseburia* abundance, which can be considered as beneficial since a reduced *Roseburia* abundance was previously associated with IBD and CRC [47,48]. The biologically relevant enrichment of members of the *Anaerostipes* (OTU52) and *Faecalibacterium* genus upon co-supplementation with GOS can also entail potential health-promoting effects. *Faecalibacterium prausnitzii* is, for example, able to exert a strong anti-inflammatory activity in the intestinal environment [49]. Moreover, increased *Faecalibacterium prausnitzii* levels have been associated with a reduction in endotoxemia in obese subjects [50]. Finally, co-supplementation of FOS showed a biologically relevant enrichment of several *Bacteroides* OTUs, the *Collinsella* genus, *Coprococcus* OTU72, *Faecalibacterium* OTU9, and an unclassified *Lachnospiraceae* species (OTU28). *Collinsella aerofaciens*, a member of the *Collinsella* genus, was previously associated with a low risk of colon cancer and is generally depleted in patients with IBD compared to healthy individuals [51,52]. The *Coprococcus* genus, on the other hand, proved to be depleted in people with depression [53]. Therefore, taking into consideration the findings of the current trial, the probiotic formulations delivered in a delayed-released capsule reported in this study could confer a number of clinically beneficial health effects in a range of patients; however, future research is needed to confirm this.

As mentioned before, intake of probiotic cells, either alone or in combination with a prebiotic, can result in a direct or indirect production of health-promoting metabolites. In the present study, the production of (b) SCFAs and ammonium was specifically assessed. SCFAs are produced mainly through saccharolytic fermentation of carbohydrates that escape digestion and absorption in the upper GIT. However, amino acid fermentation may also contribute to the production of SCFAs, mainly acetate and propionate. Moreover, if protein-derived branched-chain amino acids are fermented, this will result in the production of bSCFA [54]. Ammonium is a waste product of the urea metabolism, which is produced by urease-positive bacterial species [55]. A significantly enhanced acetate production was only observed when the probiotic digest was co-supplemented with β-glucan, GOS, or FOS. This was likely the result of the enhanced carbohydrate load being present in the colonic environment, stimulating the production of acetate by the (newly established) gut microbial communities. Acetate can be produced by many bacterial groups, including members of the *Lachnospiraceae* and *Bifidobacteriaceae* families. As summarized by Hosmer et al. [56], acetate is associated with epithelial maintenance, wound healing, and an improved gut barrier function. Moreover, acetate was previously linked with reduced colonic inflammation in mice and a protective effect against enteropathogenic colonization [56]. An enhanced propionate production was only observed when the probiotic digest was co-supplemented with GOS and FOS. Propionate can either be formed as a result of the succinate or acrylate pathway, meaning that either succinate or lactate are eventually converted to propionate [57]. Different bacterial groups are able to produce propionate, but the enhanced propionate levels could not be directly linked with one of the bacterial groups that showed a statistically significant or biologically relevant enhanced abundance. As summarized by Hosseini et al. [57], propionate was previously linked with lowered lipogenesis, serum cholesterol levels, and carcinogenesis in other tissues; as such, the enhanced propionate levels could have important clinical implications. Significantly enhanced butyrate levels were only observed for the probiotic digest alone or in combination with β-glucan. Different pathways can lead to the formation of butyrate. Acetate itself can serve as a substrate or as an essential co-substrate that needs to be consumed to complete the synthesis of butyrate from lactate [58,59]. The (direct) conversion of acetate to propionate is, amongst others, applied by members of the *Roseburia* genus [58]. The enhanced acetate production upon co-supplementation of β-glucan thus likely resulted in enhanced cross-feeding interactions between acetate-producing bacterial species and members of the *Roseburia* genus. Moreover, the enhanced *Lachnospiraceae* abundance observed for the probiotic digest alone or in combination with β-glucan likely also contributed to the increased butyrate levels as some members of this family are indeed able to produce butyrate as a result of their metabolic activity [60]. As for acetate and propionate, health-promoting effects were previously linked with enhanced butyrate levels. Butyrate can, for example, exert strong anti-inflammatory and anti-oxidative effects [61,62]. Moreover, increased butyrate levels were previously associated with an enhanced gut barrier integrity, increased satiety, and decreased weight gain [63]. Finally, the reductions in ammonium levels observed upon co-supplementation with GOS and FOS can also be considered as beneficial since markers of proteolytic fermentation, including ammonium, were previously linked with direct and indirect health effects such as cancer development [64]. The higher carbohydrate load upon administration of GOS and FOS likely outcompeted the bacterial groups or microbial pathways responsible for the breakdown of proteins. Direct linkage with one of the enhanced or reduced abundances was, however, not possible.

## 5. Conclusions

This in vitro study demonstrated that the survival of probiotic cells during the upper GIT passage largely depends on the selected probiotic formulation and administration strategy. Specifically, as probiotic bacterial species are sensitive towards the harsh environmental conditions encountered during the gastric and small intestinal passage, the use of a delayed-release capsule formulation might be recommended in order to release the probiotic bacteria at the site of their activity (distal ileum and colon) in a viable and metabolically active state. Moreover, the species/strain selection is also of high importance as the sensitivity of the probiotic cells towards the physiological conditions of the stomach and small intestine is species- and/or strain-dependent. For the commercially available delayed-release capsule tested in the current study, bacterial survival and culturability percentages of >50% were reported by the end of the upper GIT incubation, compared to <1% for the liquid, powder, and traditional capsule administration strategies. This in vitro study also indicates that the probiotic cells incorporated within the commercially available DR capsule were able to colonize the gut microbiome of the healthy adult human donors, either alone or upon co-supplementation with different prebiotic compounds (i.e., β-glucan, GOS, or FOS). Colonization was mainly observed for the *Lactobacillus* spp., which was likely in part linked with the higher survival rate of these species during the upper GIT passage as compared to the *Bifidobacterium* spp. Administration of the probiotic digest also had an effect on the gut microbial activity and community composition. More specifically, an enhanced production of SCFAs (acetate, propionate, and butyrate) and a shift in gut microbial community composition towards beneficial bacterial species such as members of the *Roseburia*, *Feacalibacterium*, *Anaerostipes*, *Collinsella*, and *Coprococcus* genus were observed. In conclusion, this study proves that elaborated testing of probiotic formulations using in vitro and/or in vivo trails is of high importance as this could aid product development and, for example, allow us to make certain claims when the product becomes available on the market.

## Figures and Tables

**Table 1:** Probiotic composition test products. DR: delayed release.

Administration Strategy	Microorganisms	In Vitro Test Dose	Corresponding Theoretical Cell Density
Liquid	*Enterococcus faecium* NCIMB 30176 *Lactobacillus acidophilus* NCIMB 30175 *Lactobacillus rhamnosus* NCIMB 30174 *Lactobacillus plantarum* NCIMB 30173	½ in vivo dose: 35 mL	5.0 × 10^9^ CFU
Powder	*Lactobacillus acidophilus* UALa-01 *Bifidobacterium bifidum* UABb-10	½ in vivo dose: 6 g	3.6 × 10^9^ CFU
Capsule	*Lactobacillus casei* *Lactobacillus rhamnosus* *Lactobacillus acidophilus* *Lactobacillus plantarum* *Streptococcus thermophilus* *Bifidobacterium breve* *Bifidobacterium longum* *Bifidobacterium bifidum* *Saccharomyces boulardii*	½ in vivo dose: 1 capsule	5.0 × 10^9^ CFU
Capsule DR	*Lactobacillus helveticus* R0052 *Bifidobacterium longum* R0175 *Lactobacillus rhamnosus* R0011 *Bifidobacterium bifidum* R0071 *Lactobacillus paracasei* Lafti L26 *Lactobacillus rhamnosus* GG *Saccharomyces boulardii ground*	1 in vivo dose: 1 capsule	65 × 10^9^ CFU

**Table 2:** Test products for the Colon-on-a-Plate™ (CoaP) experiment. ‘Blank digesta’ and ‘probiotic digesta’ refers to the upper GIT content collected at the end of the upper GIT simulation without probiotic administration and upon administration of the capsule DR, respectively.

Test Product	Content
Blank control	Blank digesta
Probiotic control	Probiotic digesta
Probiotic + β-glucan	Probiotic digesta + 3 mg/mL β-glucan
Probiotic + GOS	Probiotic digesta + 3 mg/mL galacto-oligosaccharides
Probiotic + FOS	Probiotic digesta + 3 mg/mL fructo-oligosaccharides

**Table 3:** Capsule integrity of the standard capsule and capsule DR at different time points during upper GIT passage (product, ST end, DUO end, JEJ end, and ILE end). Capsule scores are provided based on visual inspection, with the following criteria being applied: (1) capsule intact; (2) capsule damaged, but almost all probiotic content (>75%) remains in the capsule; (3) capsule damaged, but about 50% of the probiotic content remains in the capsule; (4) capsule damaged, with 25% of the probiotic content still inside the capsule; (5) capsule shell still visible, but all the product content is released; and (6) the capsule is fully dissolved. DR: delayed release.

Test Condition	Product	ST End	DUO End	JEJ End	ILE End
Capsule	1	6	6	6	6
Capsule DR	1	2	2	2	2–3

## Data Availability

The raw data supporting the conclusions of this article will be made available by the authors on request.

## Supplementary Materials

The following supporting information can be downloaded at: https://www.mdpi.com/article/10.3390/nu16162791/s1, Table S1: Non-probiotic content of the different probiotic formulations examined within this study.
