# Supplementary material for: Survival of Probiotic Bacterial Cells in the Upper Gastrointestinal Tract and the Effect of the Surviving Population on the Colonic Microbial Community Activity and Composition
Source: Nutrients. 2024 Aug 21;16(16):2791. doi: 10.3390/nu16162791 (PMC11357584; doi:10.3390/nu16162791)
Supplement: Supplementary file 1 [file nutrients-16-02791-s001.zip › nutrients-3124881-supplementary.pdf]

Table S1. Non-probiotic content of the different probiotic formulations examined within this study.

**Formulation**	**Non-probiotic content**
Liquid	*Water, extract of germinated barley, ascorbic acid, trisodium citrate, potassium sorbate*
Powder	*Organic spiruline, lecithin (95% phosphatides), organic apple powder, inulin (FOS prebiotics), organic wheatgrass juicepowder (leaf), organic alfalfa powder (leaf), organic chlorella powder, organic barley (Hordeum vulgare) leaf powder, acerola fruit juice powder extract (4:1), broccoli flower powder, Papaya (Carica papaya) fruit powder, pineapple fresh fruit concentrate (9:1), billberry fruit extract (100:1), beet root powder (fruit), rose hip (Rosa canina) fruit powder (4:1), carrot root powder, spinach leaf powder, cococa bean polyphenol extract, grape seed extract (120:1) (std. 95% OPC), green tea (Camillia sinensis) extract (leaf) (10:1), licorice root powder, Lycium berry fruit extract (4:1), ginger rhizome powder, slippery elm (Ulmus ruba) bark powder, kelp whole plant powder, alkaline pea protein isolate, citrus bioflavonoids extract, artichoke leaf extract (15:1), citric acid (anhydrous), Rhodiola (Rhodiola rosea) root dry extract (15:1), Eleuthero (Eleutherococcus senticosus) root extract (10:1), gotu kola extract (10:1), rosemary leaf extract (4:1), milk thistle seed extract (70:1), R,S alpha-lipoic acid, Ashwaganda (withania sonnifera) root extract (5:1), dandelion whole plant dry concentrate (4:1), hawthorn berry extract (10:1), beta glucans, policosanol, co-enzyme Q-10 (ubidecarenone), Stevia rebaudiana leaf powder, Astragalus (Astragalus membranaceus) root powder extract (4:1), bromelain (dietary enzyme), burdock root powder (4:1), reishi mushroom powder, shiitake mushroom powder, stevia*
Capsule	*Chicory inulin fibre (fructo-oligosaccharide), Acacia gum (fibre), bulking agent (potato starch), anti-caking agent (rice extract blend and rice fibre), coloring agent (calcium carbonate)*
Capsule DR	*Potato starch, acacia fiber, zinc oxide, ascorbic acid*
